# Effect of Active Case Finding on Prevalence and Transmission of Pulmonary Tuberculosis in Dhaka Central Jail, Bangladesh

**DOI:** 10.1371/journal.pone.0124976

**Published:** 2015-05-01

**Authors:** Sayera Banu, Md. Toufiq Rahman, Mohammad Khaja Mafij Uddin, Razia Khatun, Md. Siddiqur Rahman Khan, Md. Mojibur Rahman, Syed Iftekhar Uddin, Tahmeed Ahmed, James D. Heffelfinger

**Affiliations:** 1 International Centre for Diarrhoeal Disease Research, Bangladesh, Dhaka, Bangladesh; 2 National TB Control Program, Directorate General of Health Services, Mohakhali, Dhaka, Bangladesh; 3 Directorate of Prisons, Nazimuddin Road, Dhaka, Bangladesh; 4 Global Disease Detection Branch, Division of Global Health Protection, Center for Global Health, US Centers for Disease Control and Prevention, Atlanta, Georgia, United States of America

## Abstract

**Background:**

Understanding tuberculosis (TB) transmission dynamics is essential for establishing effective TB control strategies in settings where the burden and risk of transmission are high. The objectives of this study were to evaluate the effect of active screening on controlling TB transmission and also to characterize *Mycobacterium tuberculosis* strains for investigating transmission dynamics in a correctional setting.

**Methods:**

The study was carried out in Dhaka Central Jail (DCJ), from October 2005 to February 2010. An active case finding strategy for pulmonary TB was established both at the entry point to the prison and inside the prison. Three sputum specimens were collected from all pulmonary TB suspects and subjected to smear microscopy, culture, and drug susceptibility testing as well as genotyping which included deletion analysis, spoligotyping and analysis of mycobacterial interspersed repetitive units (MIRU).

**Results:**

A total of 60,585 inmates were screened during the study period. We found 466 inmates with pulmonary TB of whom 357 (77%) had positive smear microscopy results and 109 (23%) had negative smear microscopy results but had positive results on culture. The number of pulmonary TB cases declined significantly, from 49 cases during the first quarter to 8 cases in the final quarter of the study period (p=0.001). Deletion analysis identified all isolates as *M*. *tuberculosis* and further identified 229 (70%) strains as ‘modern’ and 100 (30%) strains as ‘ancestral’. Analysis of MIRU showed that 347 strains (85%) exhibited unique patterns, whereas 61 strains (15%) clustered into 22 groups. The largest cluster comprised eight strains of the Beijing *M*. *tuberculosis* type. The rate of recent transmission was estimated to be 9.6%.

**Conclusions:**

Implementation of active screening for TB was associated with a decline in TB cases in DCJ. Implementation of active screening in prison settings might substantially reduce the national burden of TB in Bangladesh.

## Introduction

There were an estimated 8.6 million new cases of tuberculosis (TB) and 1.3 million deaths from TB worldwide in 2012 [1]. Approximately 95% of these cases and deaths occurred in low-income countries (LICs) [1]. TB is a major cause of morbidity and mortality in prisons [2]. Health care in prison settings has historically been neglected [3]. High turnover of inmates, overcrowding, inadequate ventilation, and poor general health of many inmates facilitate the spread of infectious diseases like TB [4,5]. The reported prevalence of TB in prisons is usually much higher than in the corresponding general population [4,6]. In LICs, TB has been reported as the leading cause of death in prisons [7]. Prisons are often cited as reservoirs of TB [4] and can be a source of TB infection outside the prison environment, spreading via correctional staff, visitors, and released inmates [8]. The spread of TB from prisons to communities has been reported as a key factor in overall TB incidence, prevalence, and mortality rates [9]. Many inmates are from populations with TB risk factors such as malnutrition, poor hygiene, inadequate living conditions, and poor general health conditions [4,5] so they may have contracted TB before incarceration [7]. Once incarcerated, previously infected inmates pose a threat for transmitting TB infection and have a higher chance of progressing to active TB disease than the inmates with no history of previous infection [10,11]. Because many correctional facilities do not segregate inmates on the basis of health conditions, the spread of TB in prisons may be further facilitated.

Few studies have addressed the issue of TB in prison settings in LICs such as Bangladesh. Data on the prevalence of TB in prisons in Bangladesh are limited. Bangladesh's National Tuberculosis Control Programme (NTP) has been implementing directly observed treatment short course (DOTS) in some prisons since 2002 [12]. In Bangladesh, case finding in prison settings is generally passive and smear microscopy is conducted only for patients who seek treatment for cough and other respiratory ailments. The drug resistance pattern of these cases is not known, because culture and drug susceptibility testing (DST) for acid-fast bacilli (AFB) are not performed in correctional settings [12].

From October 2005 to December 2008, the International Centre for Diarrhoeal Disease Research, Bangladesh (icddr,b) screened all inmates at the Dhaka Central Jail (DCJ), the largest prison in Bangladesh, for symptomatic pulmonary TB. The prevalence of pulmonary TB among the inmates was 2,227/100,000 population—21 times higher than the TB prevalence in the general population in Bangladesh [12]. For a substantial proportion of cases (22%), smear microscopy results were negative but cultures were positive for *Mycobacterium tuberculosis* [12]. Multidrug resistant TB (MDR-TB) was found in 2% of confirmed cases, an alarming finding in this crowded setting, as drug-resistant *Mycobacterium tuberculosis* can be transmitted between inmates. Active screening led to early diagnosis of TB cases inside the prison which probably would not otherwise have been detected while the inmates were incarcerated. Thirty-six percent of the confirmed cases were detected within 6 months of the time that the inmates entered the prison, suggesting that a substantial number of inmates entered the prison with active TB or they were infected with *Mycobacterium tuberculosis* and developed pulmonary TB within 6 months of becoming incarcerated [12].

Several studies in different countries have suggested that in addition to the routine passive case finding strategy of self-referral, prisons need to establish active TB screening systems both at entry and throughout incarceration to control TB effectively [13]. Combining these two strategies has been successful in controlling TB in different correctional settings [14].

The lack of comprehensive molecular epidemiological data from most LICs, including Bangladesh, has limited the understanding of TB disease dynamics. There is broad variability in the genotypes of *M*. *tuberculosis* isolates from patients with epidemiologically unrelated TB, whereas the genotypes of isolates from patients who are infected by a common source are virtually identical [15,16,17]. Genotypically clustered cases of TB, defined as two or more isolates from different patients with identical genotypes, have been considered to be evidence of recent transmission [16,17]. The most frequently used genotyping methods for *M*. *tuberculosis* are restriction fragment-length polymorphism (RFLP) analysis, which targets the insertion sequence (IS) 6110 transposable element, and spoligotyping [18]. An alternative polymerase chain reaction (PCR)-based technique targeting 12 loci containing variable number tandem repeats (VNTRs) of genetic elements named mycobacterial interspersed repetitive units (MIRUs) has a discriminatory power close to that of IS6110 RFLP analysis [19,20,21,22]. The objectives of this study were to evaluate the effect of active screening on prevalence of TB and TB transmission in DCJ and determine the transmission dynamics of TB in the facility.

## Methods

### Ethics and Consent

The study protocol was reviewed and approved by the Research Review Committee and the Ethical Review Committee of International Centre for Diarrhoeal Disease Research, Bangladesh (icddr,b). We enrolled the participants in the study only after receiving informed written consent from them.

### Study Period

This study was initiated in October 2005 and continued until February 2010 in collaboration with NTP and the Directorate General of Prisons. During the initial phase of the study, between October 2005 and December 2008, active screening for symptomatic pulmonary TB was performed for current inmates inside prison but not for inmates on entry to DCJ. During this initial period, a substantial number of cases were detected within six months of the time that the inmates entered the prison, suggesting the possibility that many of the inmates entered the prison with active TB. Based on recommendations from the findings of this initial phase, active screening for symptomatic pulmonary TB on entry to DCJ was established in January 2009. Screening on entry and for current inmates was conducted from January 2009 to February 2010.

### Study Setting and Population

DCJ houses between 8,000–10,000 inmates aged ≥15 years on any given day, including 500 female inmates, although its capacity is only 2,600 inmates [12]. However, 40,000–50,000 inmates usually pass through DCJ in a typical year because of high turnover of inmates. Approximately 100–150 new inmates from throughout the country enter the facility daily. New inmates are usually kept in a common area called *amdani* (which means import) for 24 hours before being assigned to one of the 17 housing blocks based on their crimes and the duration of their sentences. Inmates who are currently on trial are generally housed with those who have already been convicted. Inmates often get released or transferred to another facility upon the direction of DCJ's administrative section without exit screening or consultation with the health service unit, even if they have been treated for medical conditions while incarcerated.

### Screening for Pulmonary TB

The methods we used for screening have been described previously [12]. In brief, two field assistants screened all new inmates (100–120/day)who were kept in the *amdani* and also current inmates (10–20/day) inside the prison cells for symptoms of active pulmonary TB. All inmates who reported having a cough of more than three weeks' duration were considered to be pulmonary TB suspects. Inmates were also screened if they had risk factors found to be associated with TB in a previous study at DCJ, such as body mass index (BMI) less than 16 kg/m^2^ [12]. Inmates with these risk factors were considered to be pulmonary TB suspects even in the absence of cough. Inmates identified as pulmonary TB suspects were immediately isolated and sent to the DCJ hospital, where a study physician examined and interviewed them to collect clinical and socio-demographic data.

**Fig 1:**
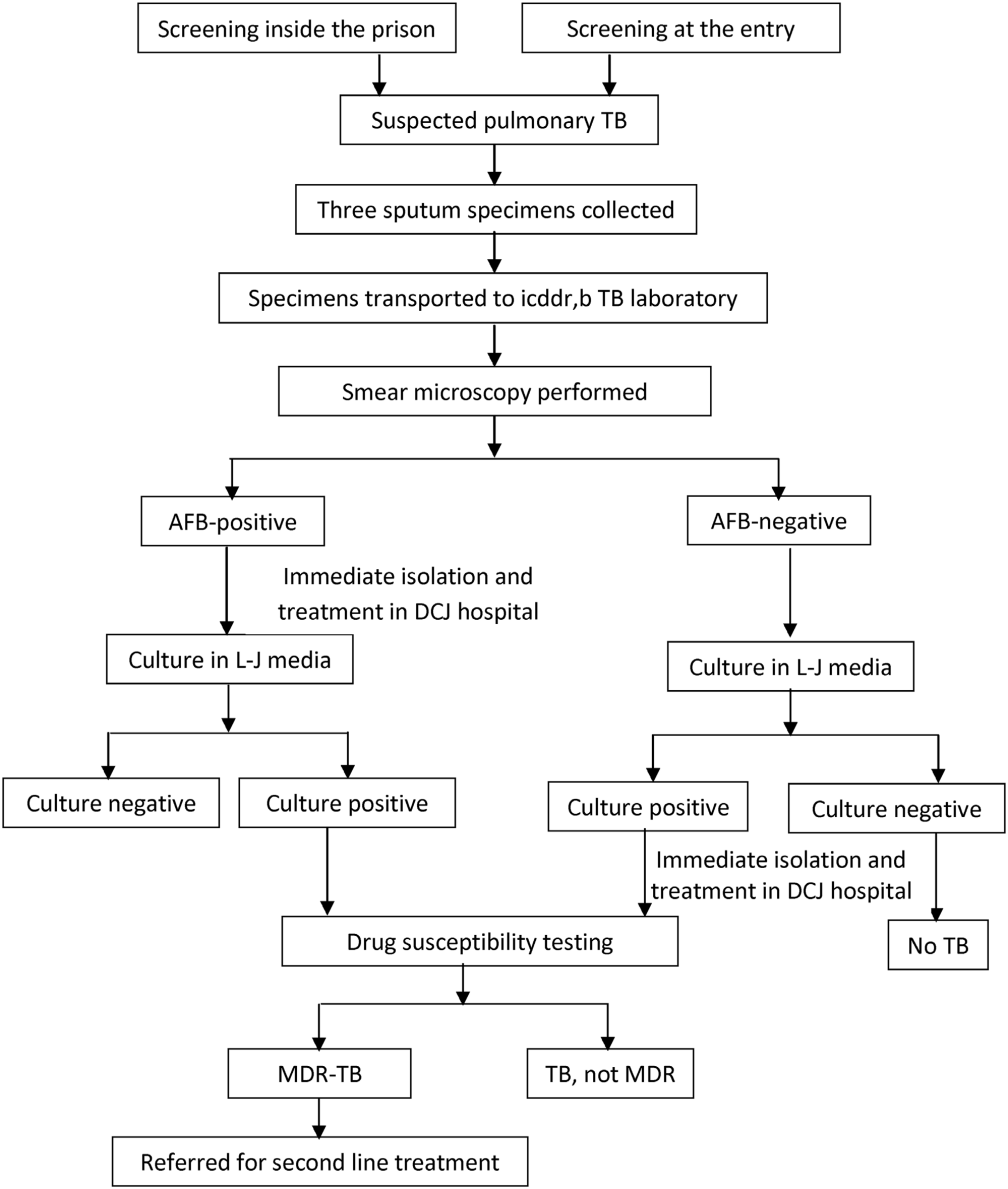
Flow chart of tuberculosis (TB) intervention at Dhaka Central Jail, October 2005 to February 2010.

Three sputum specimens were collected from each inmate with suspected pulmonary TB. The first specimen was obtained immediately after identifying the inmate as a TB suspect. The second specimen was an overnight specimen collected the next morning and the third specimen was a spot specimen, collected at the time of the second (overnight) sputum specimen. All specimens were brought to icddr,b's Tuberculosis Laboratory in an icebox carrier and processed on the same day they were collected. Smear microscopy for AFB, culture, DST, genotyping were performed. DNA fingerprinting of all the *M*. *tuberculosis* isolates was performed. All inmates with positive smears were isolated from the general DCJ population immediately after the positive results were obtained, housed in a separate TB ward, and treated according to NTP guidelines [19]. Inmates with suspected pulmonary TB who had negative results by smear microscopy were advised to contact the DCJ hospital if their symptoms became aggravated. However, inmates with negative smear microscopy results subsequently positive on culture were notified to DCJ hospital authority and also immediately isolated and treated. Inmates who were found to have MDR-TB were started on second line anti-TB treatment (Fig 1).

### Laboratory

Sputum specimens were concentrated following methods described previously [20] and smears were examined for AFB using Ziehl-Neelsen (ZN) staining under a light microscope. Sputum specimens were decontaminated following Petroffs’ NaOH method [21]. Löwenstein-Jensen (L-J) solid media was used for TB culture. The supernatant was discarded and two loopfuls of palette were inoculated on two L-J slants. These slants were incubated at 37°C for eight weeks and the inoculated slants were examined once every week for contamination as well as growth of visible mycobacterial colonies [16]. Sputum was considered culture-negative when no visible mycobacterial colonies grew on either of the L-J slants within eight weeks of incubation. *M*. *tuberculosis* from positive cultures was characterized by colony morphology and subsequently confirmed by the presence of AFB by ZN staining.

The standard proportion method was followed to test for susceptibility of *M*. *tuberculosis* isolates to isoniazid (0.2 mg/l), rifampicin (40 mg/l), ethambutol (2mg/l) and streptomycin (4 mg/l) [22]. Isolates were considered resistant to a given drug when any growth of 1% or more above the control was observed in any drug-containing quadrant plate.

Genomic DNA preparation and deletion analysis were performed as described previously [17] and spoligotyping was performed using methods previously described by Kamerbeek et al. [23] with minor modifications. PCR and calculation of MIRU copy number per locus were carried out as described previously [24,25,26]. All isolates were typed using 11 loci (2, 4, 10, 16, 23, 24, 26, 27, 31, 39 and 40) VNTR-MIRU typing. Most of the strains failed to give a PCR product with primers for the MIRU 20 locus, as seen in our previous study [26]; therefore, the results of MIRU 20 locus were excluded from this study. The rate (percent) of recent transmission was calculated by the formula: [T(c) − N(c)]/T(a) × 100, where T(c) is the total number of clustered isolates, N(c) is the number of clusters and T(a) is the total number of isolates [17,27].

### Epidemiological investigation

A cluster was defined as two or more isolates from different patients with identical spoligotype and MIRU patterns, whereas non-clustered patterns were referred to as unique. Inmates with clustered isolates were investigated by study staff using a standardized questionnaire to further establish or strengthen potential epidemiological connections in time, place and person.

### Statistical Analysis

Data were analyzed using regression models to assess the association between changes in incidence of TB from quarter to quarter (linear regression coefficient (β) with 95% confidence intervals).

## Results

A total of 60,585 inmates were screened for pulmonary TB from October 2005 to February 2010; 42,367 (70%) inmates were screened on entry to DCJ and 18,218 (30%) current inmates were screened. Four hundred sixty-six pulmonary TB cases were detected: 357 (77%) cases had positive smear microscopy results and 109 (23%) cases had negative smear microscopy results but were positive by culture (Table 1).

**Table 1:** Results of inmate screening for pulmonary tuberculosis (TB), Dhaka Central Jail, October 2005 to February 2010.

	Entry point	Prison cells	Total
**Inmates screened**	42,367	18,218	60,585
Suspected pulmonary TB cases, number (%)*	718 (2)	2,735 (15)	3,453 (6)
Confirmed pulmonary TB cases, number (%)*	44 (0.1)	422 (2)	466 (0.7)
**Samples positive for acid fast bacilli on microscopy, number (%)** ^**§**^	40 (91)	317 (75)	357 (77)
**Samples positive for TB on culture, number (%)** ^**†**^ ^**‡**^	32 (4)	414 (15)	446 (13)
**Samples negative for acid fast bacilli on microscopy but positive for TB on culture** ^**||**^	4 (9)	105 (25)	109 (23)

*Proportion among inmates screened.

^†^Proportion among suspected cases.

^‡^ Three sputum samples were collected from each of the 3,453 suspected cases for testing.

^§^ Proportion among confirmed pulmonary TB.

^||^ Proportion among confirmed pulmonary TB cases.

**Fig 2:**
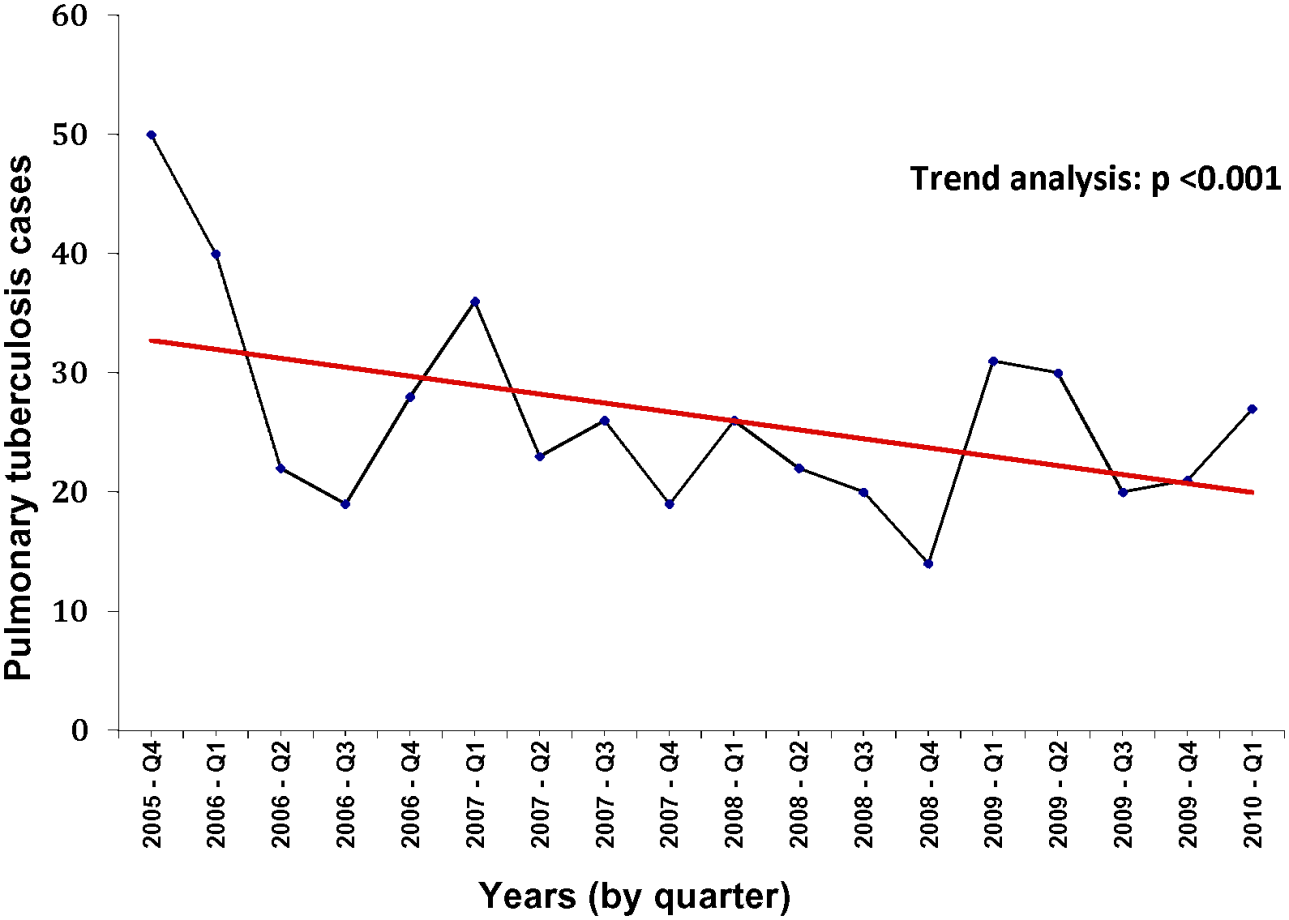
Quarterly pulmonary tuberculosis cases detected in the Dhaka Central Jail, October 2005 to February 2010.

The number of pulmonary TB cases detected each quarter at DCJ declined steadily over the study period. A total of 49 cases were detected during the first quarter of active screening and 40 cases were detected during the second quarter, after which there were only three quarters in which ≥30 cases were detected. By the final quarter of the study, only 8 cases were detected (Fig 2). A trend analysis revealed a statistically significant decline in the number of detected cases over the study period (p = 0.001).

Results of deletion analysis were available for 329 (79% of 414 culture positive TB cases among current inmates) strains of *M*. *tuberculosis* that were isolated. The RD9 region, which is strictly conserved in *M*. *tuberculosis*, was detected in all 329 tested isolates, confirming that these isolates were *M*. *tuberculosis*. Presence of the TbD1 region was observed in 100 strains (30%), indicating that these strains belonged to ‘ancestral’ or TbD1-intact *M*. *tuberculosis* lineages, while in 229 (70%) strains, the TbD1 region had been deleted, thus belonging to ΔTbD1 or ‘modern’ *M*. *tuberculosis* lineages.

A total of 373 (90% of 414 culture positive TB cases among current inmates) *M*. *tuberculosis* isolates with interpretable spoligotyping results were analyzed, revealing 135 different patterns; 51 patterns were clustered and 84 were unique. The 51 clusters contained 289 (77%) of 373 isolates (Table 2). The largest cluster comprised 91 (31%) strains belonging to the Beijing family. Thirteen percent of the isolates were of East African Indian (EAI) type [20], which corresponds to the ancestral TbD1 *M*. *tuberculosis* type [21], also named Indo-Oceanic cluster [22]. There were nine different clusters among the EAI-type strains, including EAI6_BGD1, EAI, EAI3-IND, EAI5, EAI7_BGD2 and EAI1-SOM types. The remaining 41 clusters contained 160 strains, with the number of strains in each cluster ranging from 2 to 25. Other clusters included the Central Asian strain type (CAS, CAS1 or Delhi), LAM family, U-family, T-family, H-family, X-family and MANU family strains (Table 2).

**Table 2:** Clustered *M*. *tuberculosis* strains determined by spoligotyping and distribution of spoligotyping-defined phylogenetic clades, Dhaka Central Jail, October 2005 to February 2010.

Sl	No.*	Spoligotype pattern of isolate(s)	Phylogenetic clade of isolate(s)**
1	91	▫▫▫▫▫▫▫▫▫▫▫▫▫▫▫▫▫▫▫▫▫▫▫▫▫▫▫▫▫▫▫▫▫▫▪▪▪▪▪▪▪▪▪	Beijing(1)
2	25	▪▪▪▫▫▫▫▪▪▪▪▪▪▪▪▪▪▪▪▪▪▪▫▫▫▫▫▫▫▫▫▫▫▫▪▪▪▪▪▪▪▪▪	CASI_DELHI(26)
3	16	▪▪▪▪▪▪▪▪▪▪▪▪▪▪▪▪▪▪▪▪▪▪▪▪▪▪▪▪▪▪▪▪▫▫▫▫▪▪▪▪▪▪▪	T1(53)
4	13	▪▫▫▪▪▪▪▪▪▪▪▪▪▪▪▪▪▪▪▪▪▪▪▪▪▪▪▪▪▫▪▫▫▫▫▪▪▪▪▪▪▪▪	H3 (655)
5	12	▪▪▪▪▪▪▪▪▪▪▪▪▪▪▪▪▪▪▪▪▫▫▫▫▪▪▪▪▪▪▪▪▫▫▫▫▪▪▪▪▪▪▪	LAM9(42)
6	12	▪▪▪▪▪▪▪▪▪▪▪▪▪▪▪▪▪▪▪▪▪▪▪▪▪▪▪▪▫▫▫▫▪▫▪▪▪▪▪▫▪▪▪	*EAI1_SOM (48)*
7	6	▪▪▪▪▪▪▪▪▪▪▪▪▪▪▪▪▪▪▪▪▪▪▪▪▪▪▪▪▪▪▪▪▫▫▫▫▪▪▫▫▫▫▪	T1 (244)
8	5	▪▪▪▪▪▪▪▪▪▪▪▪▪▪▪▪▪▪▪▪▫▫▫▫▪▪▪▪▪▪▫▪▫▫▫▫▪▪▪▪▪▪▪	H3-LAM9 (335)
9	5	▪▪▪▪▪▪▪▪▪▪▪▪▪▪▪▪▪▪▪▪▪▪▫▪▪▪▪▫▫▫▫▫▪▫▪▪▫▪▪▪▪▪▪	
10	5	▪▪▪▫▫▫▫▪▪▪▪▪▪▪▪▪▪▪▪▪▪▪▫▫▫▫▫▫▫▫▫▫▫▫▫▫▪▪▪▪▪▪▪	CAS(357)
11	4	▪▫▫▪▪▪▪▪▪▪▪▪▪▪▪▪▪▪▪▪▪▪▪▪▪▪▪▪▫▫▫▫▪▫▪▪▫▫▫▪▪▪▪	EAI3_IND (11)
12	4	▪▪▪▪▪▪▪▪▪▪▪▪▪▪▪▪▪▪▪▪▪▪▪▪▪▪▪▪▫▫▫▫▪▫▪▪▪▪▪▪▪▪▪	EAI5(236)
13	4	▪▪▪▪▪▪▪▪▪▪▪▪▪▪▪▪▪▪▪▪▪▫▫▫▪▫▫▫▫▫▫▪▫▫▫▫▪▪▪▪▪▪▪	H1 (283)
14	4	▪▪▪▪▪▪▪▪▪▪▪▪▪▪▪▪▪▪▪▪▪▪▫▪▫▫▫▫▫▫▫▫▫▫▪▪▫▪▪▪▪▪▪	EAI7_BGD2(96)
15	3	▪▪▪▪▪▪▪▪▪▪▪▪▪▪▪▪▪▪▪▪▪▪▫▪▪▪▪▪▫▫▫▫▫▫▪▪▫▪▪▪▪▪▪	U(1421)
16	3	▪▪▪▪▪▪▪▪▪▪▪▪▪▪▪▪▪▪▪▪▪▪▫▪▫▪▫▫▫▫▫▫▫▫▪▪▫▪▪▪▪▪▪	
17	3	▪▪▪▪▪▪▪▪▪▪▪▪▪▪▪▪▪▪▪▪▪▪▫▪▪▪▪▪▫▫▫▫▪▫▪▪▪▪▪▪▪▪▪	EAI6_BGD1(591)
18	3	▪▪▪▪▪▪▪▪▪▪▪▪▪▪▪▪▪▪▪▪▪▪▪▪▪▪▪▪▫▫▫▫▪▫▪▪▪▪▪▫▫▫▫	EAI5(138)
19	3	▪▪▪▪▪▪▪▪▪▪▪▪▪▪▪▪▪▪▪▪▪▪▪▪▪▪▪▪▪▪▪▪▪▫▫▪▪▪▪▪▪▪▪	MANU2 (54)
20	3	▪▪▪▪▪▪▪▪▪▫▫▫▫▫▫▫▫▫▫▫▫▫▫▫▫▪▪▪▪▪▪▪▫▫▫▫▪▪▪▪▪▪▪	T1 (365)
21	3	▪▪▪▫▫▫▫▪▪▪▪▪▪▪▪▪▪▪▪▪▪▪▫▫▫▫▫▫▫▫▫▫▫▫▪▪▫▫▪▪▪▪▪	CAS1_DELHI (25)
22	3	▪▫▪▪▪▪▪▪▪▪▪▪▪▪▪▪▪▪▪▪▪▪▫▪▪▪▪▪▫▫▫▫▪▫▪▪▪▪▪▪▪▪▪	EAI6_BGD1(882)
23	3	▪▪▪▪▪▪▪▪▪▪▪▪▪▪▪▪▪▪▪▪▪▫▫▫▫▫▫▫▫▫▫▫▫▫▪▪▫▪▪▪▪▪▪	EAI(1391)
24	2	▪▪▪▪▪▪▪▪▪▪▪▪▪▪▪▪▪▪▪▪▪▪▪▪▪▪▪▪▪▪▪▪▪▫▪▪▪▪▪▪▪▪▪	MANU1 (100)
25	2	▪▪▪▪▪▪▪▪▪▪▪▪▪▪▪▪▪▪▪▪▪▪▫▪▪▪▪▪▪▪▫▪▪▫▪▪▪▪▪▪▪▪▪	
26	2	▪▪▪▪▪▪▪▪▪▫▫▫▫▫▫▫▫▫▫▫▫▫▫▫▫▪▪▪▪▪▪▪▫▫▪▪▪▪▪▪▪▪▪	
27	2	▪▪▪▪▪▪▪▪▪▪▪▪▪▪▪▪▪▪▪▪▪▪▪▪▪▫▫▫▫▫▫▪▫▫▪▪▪▪▪▪▪▪▪	MANU2 (1484)
28	2	▪▪▪▪▪▪▪▪▪▪▪▪▪▪▫▪▪▪▪▪▪▪▫▫▪▪▫▫▫▫▫▫▫▫▪▪▪▪▪▪▪▪▪	
29	2	▫▪▪▪▪▪▪▪▪▪▪▪▪▪▪▪▪▪▪▪▪▪▫▪▫▪▫▫▫▫▫▫▫▫▪▪▪▪▪▪▪▪▪	
30	2	▪▪▪▫▫▫▫▪▪▪▪▪▪▪▪▪▫▪▫▫▫▫▫▫▫▫▫▫▫▫▫▫▫▫▪▪▪▪▪▪▪▪▪	
31	2	▪▪▪▪▪▪▪▪▪▪▪▪▫▪▫▫▪▪▪▪▪▪▪▪▪▪▪▪▪▪▪▪▫▫▫▫▪▪▪▪▪▪▪	T3 (1655)
32	2	▪▪▪▫▫▫▫▫▫▫▫▫▪▪▪▪▪▫▪▪▪▪▪▪▪▪▪▪▪▪▪▪▫▫▫▫▪▪▪▪▪▪▪	X3 (92)
33	2	▪▪▪▪▪▪▪▪▫▫▫▪▪▪▪▪▪▪▪▪▫▫▫▫▪▪▪▪▪▪▪▪▫▫▫▫▪▪▪▪▪▪▪	LAM3(33)
34	2	▪▪▪▪▪▪▪▪▪▪▪▪▪▪▫▫▫▪▫▫▫▫▫▫▪▪▪▪▪▪▪▪▫▫▫▫▪▪▪▪▪▪▪	T1 (766)
35	2	▪▪▪▪▪▪▪▪▪▪▪▪▪▪▪▪▪▪▪▪▪▪▪▪▪▪▪▪▪▪▫▪▫▫▫▫▪▪▪▪▪▪▪	H3 (50)
36	2	▪▪▪▪▪▪▪▪▪▪▪▪▪▪▫▪▪▪▪▪▪▪▪▪▪▪▪▪▪▪▫▪▫▫▫▫▪▪▪▪▪▪▪	H3 (75)
37	2	▪▪▪▪▪▪▪▪▪▪▪▪▪▪▪▪▪▪▪▪▪▪▪▪▪▫▫▫▫▫▫▪▫▫▫▫▪▪▪▪▪▪▪	H1 (47)
38	2	▪▫▫▪▪▪▪▪▪▪▪▪▪▪▪▪▪▪▪▪▪▪▪▪▪▪▪▪▪▪▫▫▫▫▫▫▪▪▪▪▪▪▪	
39	2	▪▪▪▪▪▪▪▪▪▪▪▪▪▪▫▪▪▪▪▪▪▪▫▪▪▪▪▫▫▫▫▫▪▫▪▪▫▪▪▪▪▪▪	
40	2	▪▪▪▪▪▪▪▪▪▪▪▪▪▪▪▪▪▪▪▪▪▫▫▫▫▪▫▫▫▫▫▫▫▫▪▪▫▪▪▪▪▪▪	
41	2	▪▪▪▪▪▪▪▪▪▪▪▪▪▪▪▪▪▪▪▪▪▪▫▪▪▪▪▪▫▫▫▫▪▫▫▫▫▪▪▪▪▪▪	
42	2	▪▫▪▪▪▪▪▪▪▪▪▪▪▪▪▪▪▪▪▪▪▪▫▪▫▫▫▫▫▫▫▫▫▫▪▪▪▫▪▪▪▪▪	
43	2	▪▪▪▪▪▪▪▪▪▪▪▪▪▪▪▪▪▪▪▪▪▪▪▪▪▪▪▪▫▫▫▫▪▫▪▪▪▪▪▪▫▫▫	
44	2	▪▫▫▪▪▪▪▪▪▪▪▪▪▪▪▪▪▪▪▪▪▪▪▪▪▪▪▪▫▫▫▫▪▫▪▪▪▪▪▫▫▫▫	
45	2	▪▪▪▪▫▫▪▪▪▪▪▪▪▪▪▪▪▫▪▪▪▪▪▪▪▪▪▪▪▪▪▪▫▫▫▫▫▫▫▫▫▫▫	
46	2	▪▪▪▫▫▫▫▪▪▪▪▪▪▪▪▪▪▪▪▪▪▪▫▫▫▫▫▫▫▫▫▫▫▫▫▫▫▫▫▫▫▫▫	CAS (1264)
47	2	▪▪▪▪▪▪▪▪▪▪▪▪▪▪▪▪▪▪▪▪▪▪▫▪▪▪▪▪▫▫▫▫▪▫▪▪▫▪▪▪▪▪▪	EAI6_BGD1 (292)
48	2	▪▪▪▫▫▫▫▫▫▫▫▫▪▪▪▪▪▪▪▪▪▪▪▪▪▪▪▪▪▪▪▪▫▫▫▫▪▪▪▪▪▪▪	T1 (344)
49	2	▪▪▪▪▪▪▪▪▪▪▪▪▪▪▪▪▪▪▪▪▪▪▪▪▪▪▪▪▫▫▫▫▫▫▪▪▪▪▪▪▪▪▪	U (458)
50	2	▪▫▫▪▪▪▪▪▪▪▪▪▪▪▫▪▪▪▪▪▪▪▪▪▪▪▪▪▪▪▫▪▫▫▪▪▪▪▪▪▪▪▪	
51	2	▪▪▪▪▪▪▪▪▪▪▪▪▪▪▪▫▫▫▪▫▫▫▫▫▫▪▪▪▪▪▪▪▪▫▫▪▪▪▪▪▪▪▪▪	
Total strains	289		

NOTE: Vertical bars correspond to spoligotype (ST) clusters. Filled square indicates “spacer present” in the drug resistant region, non-filled square means “spacer deleted”.

* Number of isolates with the same spoligotype;

** Where shown, numbers in brackets indicate the "Shared-type number (SIT) of the SpolDB4 / SITVIT database, in case the corresponding spoligotype was not represented in SpolDB4, the field was left empty.

Genotyping was performed on 408 isolates using MIRU typing and revealed 369 different patterns; 22 patterns were clustered and 347 were unique. The 22 clusters contained 61 (15%) isolates (Table 3).

**Table 3:** Sizes and numbers of tuberculosis (TB) clusters and total numbers of inmates in TB clusters by size of cluster, Dhaka Central Jail, October 2005 to February 2010.

Cluster size (number of inmates with pulmonary TB)	Number of clusters	Total number of inmates in clusters
**2**	14	28
**3**	5	15
**4**	1	4
**6**	1	6
**8**	1	8
**Total**	22	61

**Fig 3:**
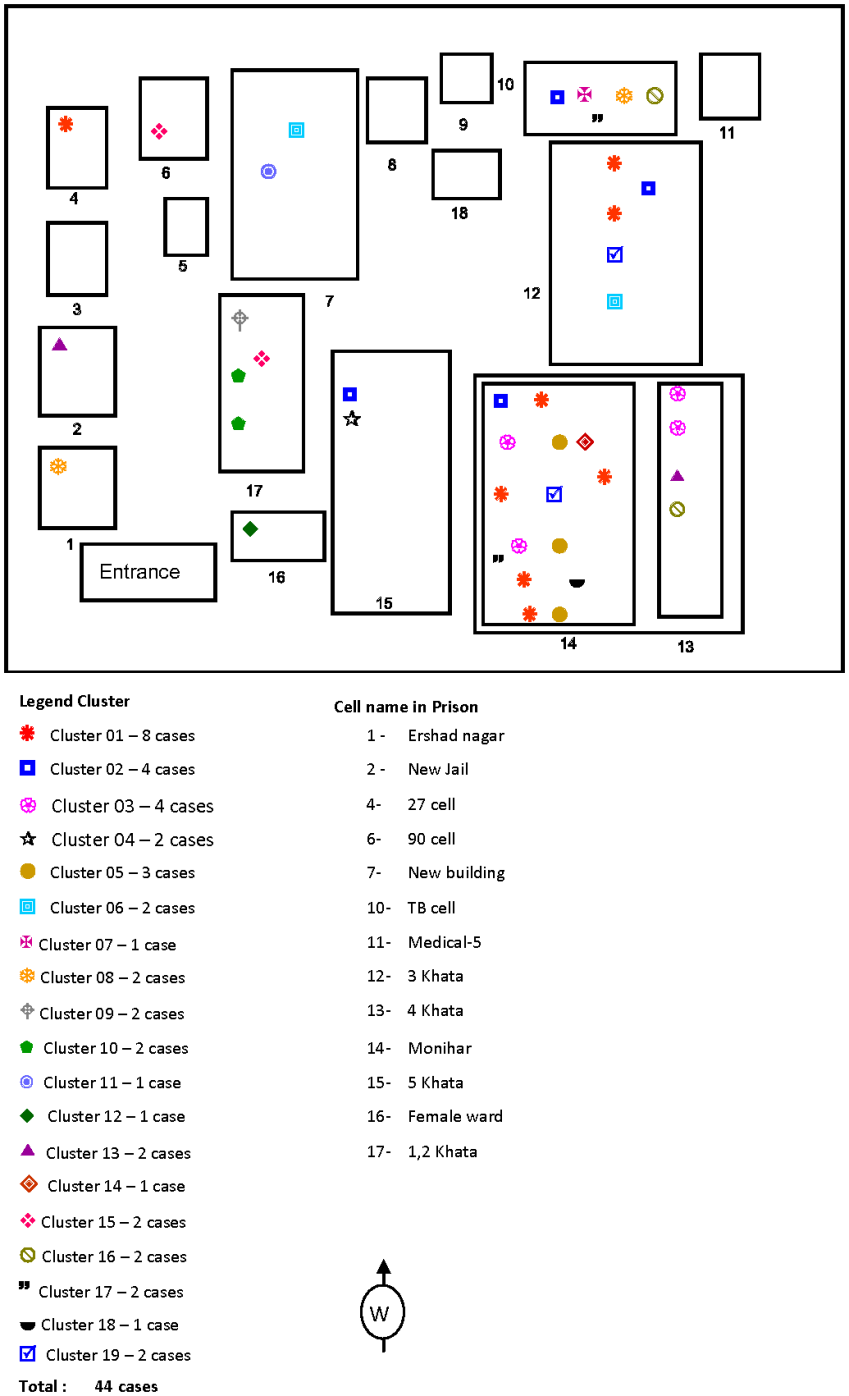
Diagram of Dhaka Central Jail showing locations of mycobacterial interspersed repetitive units (MIRU) clusters, October 2005-November 2007.

**Fig 4:**
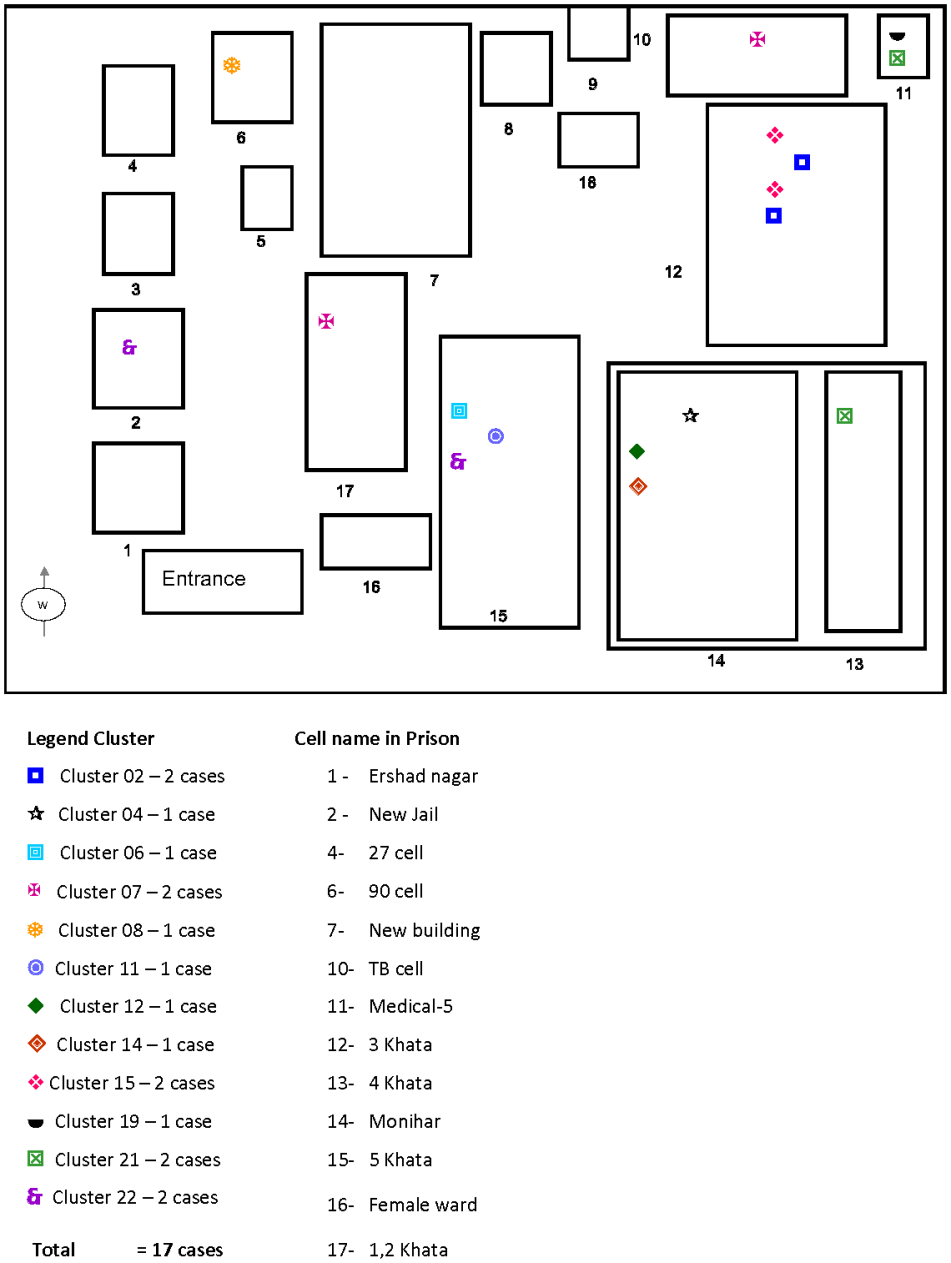
Diagram of Dhaka Central Jail showing locations of mycobacterial interspersed repetitive units (MIRU) clusters (December 2007 to February 2010).

The largest cluster comprised eight isolates, all of which belonged to the Beijing family. Among the other clusters, one comprised six isolates, one comprised four isolates, five comprised three isolates and the remaining fourteen clusters comprised two isolates each. Clustering was high among the modern strain isolates as out of the 329 isolates analyzed, all of the 47 isolates that were clustered fell into the modern lineage (data not shown). The 91 isolates classified as Beijing strains by spoligotyping could be classified into 76 distinct types using VNTR-MIRU typing; 7 of these were clustered and 69 were unique. Inmates with isolates that had similar MIRU patterns had medical and epidemiological data reviewed to identify any epidemiological links. Apparent epidemiological links were found among the clustered cases as all the clustered cases either lived in the same prison cells or shared common spaces within the prison (Figs 3 and 4).

We analyzed the clustered strains to determine the transmission pattern over the study period. The calculated rate of recent transmission was 9.6% during the entire study period, where T(c) = 61, N(c) = 22 and T(a) = 408. Clustering was higher during the first 26 months of the study when 39 (15%) of 258 isolates comprised 14 different clusters (Fig 3) and the rate of recent transmission was 9.7% compared to the last 27 months of the study when 10 (7%) of 150 isolates comprised 5 different clusters (Fig 4) and the rate of recent transmission was 3.3% (p < 0.05). The transmission rate was high in “Monihar” and “3 Khata” prison cells (Fig 3) which housed a large number of inmates and inmates with long histories of imprisonment.

DST was performed on 444 of the 446 isolates obtained on culture; two specimens were not available for DST because of contamination. Three hundred nine (70%) isolates available for DST were susceptible to all first-line anti-tubercular drugs and 135 (30%) isolates were resistant to ≥1 first-line drug. Among the resistant isolates, 11% were resistant to isoniazid, 2% were resistant to rifampicin, 8% were resistant to ethambutol, and 24% were resistant to streptomycin. Overall, 8 (2%) isolates were MDR-TB. There was no association between presence of clustering and drug resistance. However, drug resistance was significantly higher among isolates in the ancestral than in the modern lineage.

## Discussion

We found that the TB case burden decreased significantly in DCJ over the study period, possibly a result of active case finding and immediate isolation and treatment when a pulmonary TB case was detected. Clustering of cases also decreased over the study period which indicates that active case finding and immediate isolation and treatment of persons with pulmonary TB resulted in decreased transmission in DCJ.

In LIC countries like Bangladesh, where the burden and risk of TB disease are the greatest, data on molecular epidemiology related to transmission of TB are scarce. Studies of transmission dynamics are based upon the assumption that transmission has occurred recently when patients are found to be infected with isolates with identical molecular fingerprints, whereas those infected with isolates with unique fingerprint patterns are presumed to represent transmission further back in time and are thus considered to result from reactivation of strains [28]. The incubation period of TB disease varies from a few months to many years, so in order to get a clear picture of transmission patterns in a closed area it is important to obtain fingerprints of TB strains collected from all TB cases in that area for several years. Little data exist on this kind of transmission study in prison populations on this subcontinent and to our knowledge this is the first study of its kind.

In this study, characterization by deletion analysis using RD9 revealed that all the isolates investigated were *M*. *tuberculosis*. TbD1 analysis revealed that the majority of the isolates (70%) belonged to modern *M*. *tuberculosis* strains and 30% of samples belonged to ancestral strains because they belonged to a lineage of strains that separated from all other *M*. *tuberculosis* strains before the deletion of TbD1 occurred [27]. The percentage of modern strains in this study is similar to what we found in a previous study at a tertiary care hospital in Dhaka in which 69% of isolates were from modern strains [16]. However, it differs from a study conducted in a rural area of Bangladesh in which only 35% of isolates were from modern strains [17]. One study found that contacts exposed to persons infected with modern strains have a three-fold higher risk of developing active disease [29], suggesting that more than two-thirds of DCJ inmates with pulmonary TB had been exposed to strains with increased virulence. Exposure to modern strains places individuals at increased risk for infection because of the increased potential for dissemination of TB [30]. The virulent nature of modern strains and risk factors associated with prisons make TB control more challenging. Therefore, it would be important for NTP to impose a more effective TB control program in correctional settings to avoid TB outbreaks in these settings. We also documented that *M*. *tuberculosis* Beijing type is the predominant phylogenetic lineage in DCJ, accounting for 31% of investigated strains. Similarly, a study conducted at a tertiary referral hospital showed that the predominant strains (19%) were of the Beijing family [16]. On the other hand, a study from rural Bangladesh showed that *M*. *tuberculosis* EAI is the predominant lineage (25%) [17].

One of our objectives was to investigate the TB transmission pattern in this prison setting. It was difficult to analyze the transmission pattern among all or even most of the cases as many inmates with confirmed pulmonary TB were transferred to other facilities or released from DCJ, similar to what has been found in other studies in correctional facilities in different parts of the world [2]. We identified 22 clusters that contained 15% of the total cases found. A considerable proportion of *M*. *tuberculosis* isolates obtained were clustered and there was an apparent epidemiological link among all the clustered cases as all the clustered cases either lived in the same prison cells or shared common spaces within the prison. The clustered strains were found across different prison cell blocks, perhaps because inmates share some common spaces during the day, though they have designated cells. The transmission rate was high in two of the prison cells (Fig 3) which housed a large number of inmates and inmates with long histories of imprisonment. However, it was difficult to determine the source case given the high turnaround in the prison population. The rate of recent transmission was 9.5%, which is relatively high in comparison to our previous study in a rural setting which was 6.5% [17]; the high transmission rate underscores the risk of TB transmission in a confined setting like DCJ. Continued active screening in the prison and the introduction of active entry screening coincided with a declining trend in both TB case burden and transmission, probably resulting from detection of inmates with active TB earlier than they might have been without active screening and immediate isolation and initiation of anti-TB treatment.

We acknowledge the limitation of our study, as we did not have any control prison setting and also there was no baseline data of TB case burden in DCJ before the start of our intervention. It is difficult to conclude that our interventions led to a decrease in TB burden as well as transmission in the absence of a control group (where intervention was not done) and strong baseline data. However, the national data suggests that, in Bangladesh there was a steady increase in the absolute number of TB cases notified during the last decade (2004–2013) with stable notification rate [31]. This national data favors our study findings which indicated the decline in TB case burden of DCJ possibly resulted from our interventions.

Our findings raise interesting questions about TB transmission dynamics in this setting and have important implications for TB control in prisons. Several studies have shown high rates of transmission of drug resistant strains in prison settings [32,33]. The emergence of drug resistant TB poses a serious threat for prison settings. In our study, 2% of inmates had MDR-TB, which is comparable to the rate in the general population in Bangladesh [1]. Measures need to be taken now to address transmission of drug-resistant in the corrections system. Treatment of drug resistant TB is challenging in the community and in correctional settings and transmission of drug-resistant TB strains to people probably results in an increase in the time that they remain infectious compared to the amount of time people remain infections following transmission of drug-susceptible TB strains [32,33].

We screened inmates for symptoms suggestive of pulmonary TB but it is likely that we failed to detect some pulmonary TB cases. The use of chest radiography for inmates with symptoms would have enhanced detection of pulmonary TB, particularly among the inmates with pulmonary TB who had negative smears on microscopy—these persons constituted almost one-quarter of inmates with pulmonary TB in this study. Earlier TB detection would have resulted in earlier isolation and treatment, further limiting TB transmission in DCJ.

Delayed diagnosis, incomplete treatment resulting from repeated prison transfers or poor linkage between prison and community DOTS systems, and conditions like overcrowding, poor ventilation and lack of infection control measures encourage the transmission of TB in prisons. Several TB outbreaks resulting from transmission involving both inmates and prison staff have been reported in different prison settings across the world [34,35,36]. Therefore, it is of utmost importance to understand the transmission dynamics in these confined settings where close physical proximity of inmates and long duration of exposure can lead to high prevalence of TB if persons with active TB cases are not found, isolated, and treated as early as possible. Urgent action is needed to prioritize the implementation of TB control strategies adopted by the World Health Organization in prison settings to ensure the TB control efforts in correctional facilities are adequate and comparable with those implemented in communities to reduce the global burden of TB.
